# A Family of Novel Cyclophilins, Conserved in the Mimivirus Genus of the Giant DNA Viruses

**DOI:** 10.1016/j.csbj.2018.07.001

**Published:** 2018-07-11

**Authors:** Sailen Barik

**Affiliations:** EonBio, 3780 Pelham Drive, Mobile, AL 36619, USA

**Keywords:** Megavirus, Mimivirus, Klosneuvirus, Chaperone, Protein prolyl isomerase, Cyclophilin, Amoeba

## Abstract

The cyclophilin (abbreviated here as CYN) family represents a large group of protein prolyl isomerase (PPIase), many of which are also chaperones that promote proper folding of a large variety of client proteins. Over the past few years, megaviruses with giant DNA genomes were discovered and placed in the order *Megavirales*. Recently, the first complete genome sequence of *Acanthamoebaae polyphaga* mimivirus, a member of the *Mimiviridae* family of the *Megavirales* order, revealed a novel CYN that lacked PPIase activity and contained unique peptide insertions. To examine the universality of this unique CYN, I have reviewed and compared all CYN sequences found in the *Megavirales* genomes that are currently available. The results showed that multiple unique sequence features are indeed highly conserved in CYNs of all members of the *Mimivirus* genus, whereas viruses of the other genera of this family encode canonical CYNs only. Overall, the primary structures of all Mimivirus CYNs were highly similar, but different from those in the other genera, although the higher order structures were conserved across genera. In summary, this review establishes a family of novel but conserved cyclophilins that occur in a single viral genus.

## Highlights

• Cyclophilins (CYNs) are ubiquitous cellular protein prolyl isomerases.
• Recently, the CYN genes have been found in giant viruses (megaviruses) as well.
• All megaviral CYNs are of two kinds, the cellular type and the unique ones.
• The unique CYN family contains multiple novel features.
• These findings will promote investigations of megavirus phylogeny and evolution.

## 1 Introduction

**Fig. 1:**
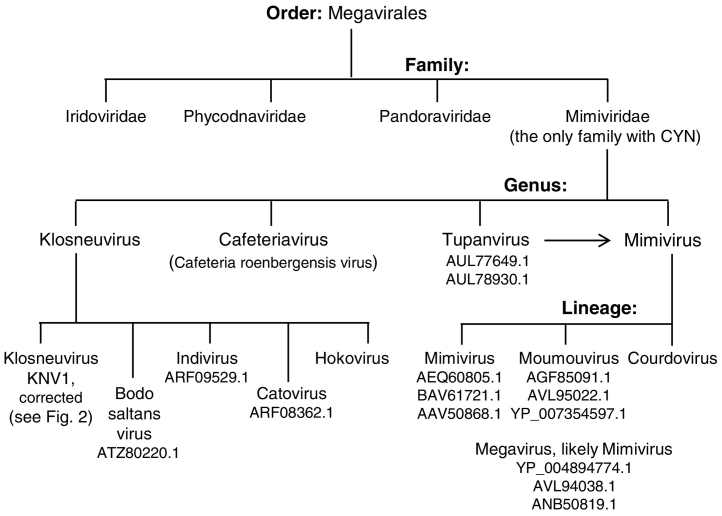
Classification of select viruses in the *Megavirales* order. This chart is not meant to be exhaustive, but represents only a subset of families, relevant to this study. Thus, *Poxviridae* and *Asfarviridae* were excluded, and so was the recently discovered Marseillevirus, which likely represents a new family of giant viruses [15]. The corrected sequence of KNV1 CYN has been described (Fig. 2). Each GenBank number represents a nonredundant CYN; absence of a GenBank number under a virus name indicates that no CYN homolog was found in that viral genome. CYN sequences from Mimivirus Bombay (AMZ03048.1) and Hirudovirus strain Sangsue (AHA45239.1) were also excluded, because they were essentially identical (100% and 99% amino acid identity, respectively) to that of the *Acanthamoeba polyphaga* mimivirus (AAV50868.1). Isolates that were originally called Tupanvirus and Megavirus have been placed under the Mimivirus designation. As stated earlier, new megaviruses are being discovered and classified [13, 14, 16], some of which may eventually be assigned into a lineage of *Mimiviridae*. Examples of such viruses, not placed in this classification tree, include: Terra2, *Aureococcus anophagefferens* virus (AaV) (“brown tide virus”), *Pyramimonas orientalis* virus, *Phaeocystis globosa* virus (PgV) (represented by PgV-16 T strain), Organic Lake Phycodnavirus 1 and 2 (hosts of Organic Lake virophage), Yellowstone Lake Phycodnavirus 4 (YSLGV4), and *Chrysochromulina ericina* virus (CeV) (e. g. CeV 01).

Cyclophilins are a major class of immunophilins, possessing protein prolyl isomerase (PPIase) activity that accelerates the *cis-trans* isomerization of the proline peptide bonds in the unstructured polypeptide [[1], [2], [3], [4], [5], [6], [7]]. They also facilitate native folding of their polypeptide substrates through the PPIase activity as well as chaperone function [8, 9]. Of note, I have used the recently accepted root, CYN, for cyclophilin (instead of the older, CyP), as described previously [10, 11]. The cyclophilins (CYNs) are ubiquitously present in all eukaryotes ranging from advanced metazoans to unicellular protists such as fungi, protozoan parasites and amoeba; in contrast, they have been absent in traditional viral genomes, which are much smaller than cellular genomes. However, the recent discovery of very large viruses (often broadly called “megaviruses”), which contain double-stranded DNA genomes in the megabase range, has obfuscated the difference between viruses and cells [[12], [13], [14]]. The new and expanding *Megavirales* order, created to accommodate these viruses (Fig. 1), houses at least four established megavirus families, which are further divided into several genera. Many of these giant viruses encode genes that were formerly in the cellular realm, such as those related to translation [12, [17], [18], [19], [20], [21], [22]]. Pioneering study of one of the first sequenced megavirus genomes, that of the *Acanthamoebaae polyphaga* Mimivirus [21], led to the discovery of a mimiviral protein that was structurally similar to CYN but contained several unique sequence features and properties, notably, peptide insertions, lack of PPIase activity, CsA insensitivity, and formation of multimers [23]. In view of the potentially critical role of CYN in megavirus life cycle and host-virus interaction, I considered it important to investigate the occurrence and nature of cyclophilins in these newly discovered viruses. To this end, I have explored the available genome sequences of all megaviruses for their CYN orthologs, which revealed conserved genus-specific CYN subsets. Specifically, the unique CYN sequences were found exclusively in the *Mimivirus* genus, whereas the sister *Klosneuvirus* genus encoded canonical CYN sequences only. These findings may have implications in the mechanism of evolution and functional specificity of viral cyclophilins.

## 2 The *Megavirales* Order of Viruses

Since the giant viruses are relatively new, in order to have a roadmap for this study I first collected information on all major viruses in *Megavirales* order, and here, I present them in their currently accepted taxonomic ranks (Fig. 1). Only selected families of the parent order are shown, of which *Mimiviridae* is the most populated and best studied. All genomes were mined for CYN-like sequences in multiple ways, as described in Methods. The three other families (*Iridoviridae, Phycoviridae, Pandoraviridae*) contained no identifiable CYN sequence. The *Mimiviridae* family is divided into four Genera, although the recent trend is to view *Tupanvirus* as a subset of *Mimivirus* genus. The two most populated genera are *Klosneuvirus* and *Mimivirus*, both of which are further subdivided into several “lineages”, as shown (Fig. 1). No CYN-like sequence was found in the single known member of the *Cafeteriavirus* genus [24] and in the Hokovirus and Courdovirus lineages. It should be mentioned that new megaviruses continue to be discovered, and as such, this is an emerging field in which the taxonomic position of many viruses remains to be finalized. Also to note that the extended *Mimiviridae* family is sometimes referred to as *Megaviridae*, although the latter has not been recognized by ICTV (International Committee on Taxonomy of Viruses). Thus, to avoid confusion and redundancy, I elected the Mimivirus genus to accommodate three recent viruses that have been called “Megavirus” (Fig. 1) [17, 25].

## 3 Cyclophilin Sequences in the *Mimiviridae* Family of Giant Viruses

**Fig. 2:**
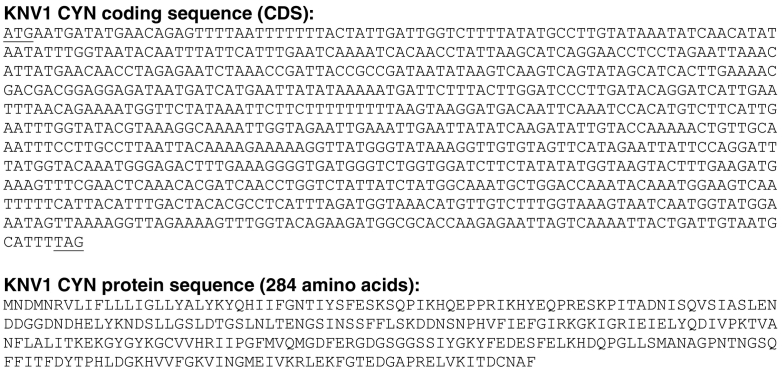
Nucleotide sequence and conceptually translated protein sequence of Klosneuvirus KNV1 cyclophilin. The correction of the sequence has been described in the text. This is an intronless gene, and the start and stop codons are underlined.

As observed above, *Mimiviridae* is the only family in the *Megavirales* order that encode CYNs (Fig. 1). Within this family, and if we accept Tupanviruses as essentially Mimiviruses, there are only two genera that encode CYNs, namely *Klosneuvirus* and *Mimivirus*. As seen by the GenBank accession numbers, these two genera contain, respectively, 4 and 11 viruses, leading to a total of 15 CYN orthologs in the *Megavirales* order. Regarding the Klosneuvirus KNV1, its 101-amino acid long cyclophilin sequence in the GenBank (Accession ARF11440) lacks approximately 183 residues at the C-terminus that is highly conserved in this family. This appears to be due to an extra T in the homopolymeric stretch of 8 T's in the coding sequence, leading to a premature stop codon. We do not know whether this is a sequencing error, common in homopolymeric nucleotide runs, or a naturally occurring truncated CYN. Nevertheless, deletion of this extra T from the *Klosneuvirus* genome sequence (GenBank: KY684108.1) generated a coding sequence, the conceptual translation of which produced a 284-residue CYN protein sequence (Fig. 2) that is highly similar to the other *Mimivirus* CYNs and has been used in the rest of the review.

## 4 Distinctive Primary Structures of Klosneuvirus and Mimivirus Cyclophilins

**Fig. 3:**
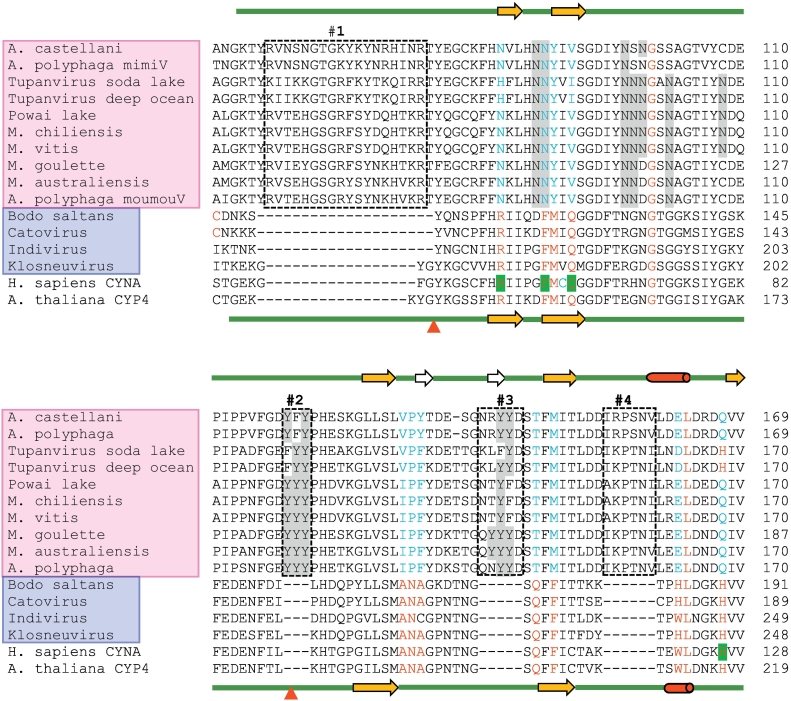
Distinctive sequence features of the *Klosneuvirus* and *Mimivirus* cyclophilins. Retrieval of the sequences and multiple alignment have been described in detail under Methods. Only the relevant portion, important for PPIase catalytic activity, is shown. For space constraints, the virus names are written in arbitrary shorthand, since no abbreviated nomenclature system has yet been established for the giant viruses. The full GenBank names of the viruses, the corresponding accession numbers (also in Fig. 1), and total number of amino acids (aa) in the full-length protein are as follows (from top to bottom): *Acanthamoeba castellanii* mamavirus (AEQ60805.1; 234 aa); *Acanthamoeba polyphaga* mimivirus, Kasai strain (AAV50868.1; 234 aa; the sequence BAV62707.1 of the *shirakomae* strain was identical, and therefore, excluded); Tupanvirus soda lake (AUL77649.1; 245 aa); Tupanvirus deep ocean (AUL78930.1; 249 aa); Powai lake megavirus (ANB50819.1; 245 aa); *Megavirus chiliensis* (YP_004894774.1; 246 aa); *Megavirus vitis* (AVL94038.1; 244 aa); *Moumouvirus goulette* (AGF85091.1; 262 aa); *Moumouvirus australiensis* (AVL95022.1; 242 aa); *Acanthamoeba polyphaga* moumouvirus (YP_007354597.1; 242 aa); Bodo saltans virus (ATZ80220.1; 228 aa); Catovirus CTV1 (ARF08362.1; 226 aa); Indivirus (ARF09529.1; 286 aa); Klosneuvirus (likely 284 aa; see Fig. 2). Cellular *Homo sapiens* cyclophilin A (P62937.2; 165 aa) and *Arabidopsis thaliana* CYP4 (NP_001154684; 313 aa) are also included for comparison. The Mimivirus and Klosneuvirus orthologs are boxed in pink and purple, respectively, and the inserts in Mimivirus sequences are also boxed by dotted lines and numbered #1 to #4. The secondary structural elements of the two groups are drawn respectively on top and bottom: orange arrow (β-strand), red cylinder (α-helix); green line (linker sequences outside helices and strands). Two Mimivirus-specific short helices are shown as white arrows. The 13 consensus residues, important for PPIase activity [26], are in red colour; those that are different from consensus are in teal colour. Mutation of the green highlighted residues in human CYNA (R55, F60, Q63, H126) into Ala destroyed PPIase activity but not chaperone activity [9]. The two red triangles are common sites of peptide insertion in a variety of cyclophilins (detailed under Discussion). Asn (N) and Tyr (Y), relatively abundant in several loop regions, are highlighted in grey.

**Fig. 4:**
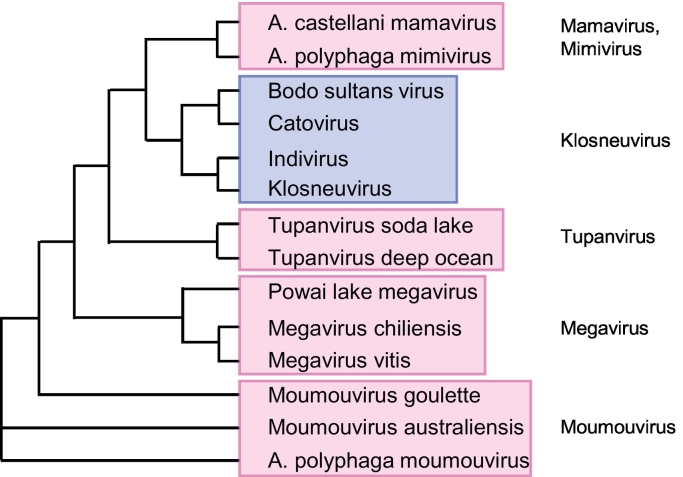
Phylogenetic tree of the giant virus CYNs. The CYN polypeptide sequences were compared by multiple alignment using Clustal Omega, and the phenogram tree was generated as described previously [10]. Virus names are described in detail in Fig. 3; the mimivirus and related clusters are boxed in pink, and the klosneuvirus group, in purple.

Next, I examined the relationships among the CYNs by multiple sequence alignment; to conserve space, only the enzymatically important area is presented here (Fig. 3). For comparison, I also included an animal (human) and a plant (*Arabidopsis thaliana*) CYN as prototypes of canonical CYN sequences. It was immediately obvious (Fig. 3) that all Mimivirus CYN sequences are highly similar, and as a result, clustered together. The Klosneuvirus CYN sequences were also highly similar, but differed significantly from the Mimivirus group. Within each group, the small subsets were also obvious, as shown in a phenogram plot of sequence similarity, where they are clustered together (Fig. 4). For the sake of brevity, I will often refer to these two groups as Klosneuvirus CYN (klosCYN for short) and Mimivirus CYN (mimiCYN for short).

In general, the mimiCYN group followed the uniqueness noted in the first Mimivirus CYN characterized [23], whereas the klosCYN group was canonical, resembling human CYNA (commonly known as hCyPA) and Arabidopsis CYN (commonly CyP4). As shown (Fig. 3), the 13 major amino acid residues that are essential for the PPIase enzymatic activity of canonical CYN [26] are all conserved in the klosCYN group, with the minor exception of Indivirus CYN, in which Ala224 is replaced by Cys. In sharp contrast, with few exceptions, all of the 13 residues in the mimiCYN family differed from the canonical ones (Fig. 3), the most common exception being the single conserved Gly (number 100 in the majority of the mimiCYNs). Indeed, the *A. polyphaga* CYN, a representative mimiCYN, showed a total lack of PPIase activity when assayed in vitro [23].

Lastly, the catalytically important area of the mimiCYN sequence, as shown (Fig. 3), also contains four unique insertions (boxed and numbered in Fig. 3), including the large insert #1, 18-residues in length. Also to note that the mimiCYNs tend to have areas rich in specific amino acids, some of which are highlighted in Fig. 3; they include Asn-rich areas between inserts #1 and #2, and Tyr dimers and trimers within inserts #2 and #3, mostly located in the loop regions in the conserved secondary structure (described below).

## 5 Conserved Secondary Structures in all Megavirales Cyclophilins

In the face of these major differences in the primary structure, comparison of the higher order structures of the two CYN groups was called for. To this end, I compared the two major secondary structural elements, namely α-helix and β-strand, of these sequences (Fig. 3). The klosCYN structure was adopted from the archetypal human CYNA, and the mimiCYN structure from the previous publication [23], both of which are shown schematically, respectively above and below the sequence alignment (Fig. 3). The bioinformatically predicted secondary structures (not shown) of all CYN sequences, obtained with SABLE software, were essentially identical to these group-specific structural elements, thus adding confidence to the generalization, the only difference being the two short α-helices in the Mimivirus group (Fig. 3). Together, they confirm and extend the previous finding that the X-ray crystal structures of human CYNA and *A. polyphaga* mimivirus CYN were highly superimposable [23]. These results clearly show that the mimiviral CYNs retain CYN-like structural folds in spite of their significant departure from the canonical amino acid sequence, which reciprocally justifies their cyclophilin designation. The large insertions in the mimiCYNs apparently did not affect the structural folds because they were located in the flexible linker areas (green lines in Fig. 3), as the crystal structure of *A. polyphaga* CYN also revealed [23]. Insert #3 was the only insert that was not located in a canonical CYN linker, but it created a short β-strand of unknown significance and with no obvious effect on the rest of the molecule.

## 6 Methods

All sequences were obtained from GenBank, and homology searches with various CYN query sequences, including those of the large DNA viruses, were conducted with the appropriate protein BLAST program, including PSI-BLAST, PHI-BLAST and DELTA-BLAST using default parameters. Searches were performed genomewide as well as within a subset by specifying the organism in the pull-down list, such as “Mimiviridae”, “Mimivirus”. Host organism names, such as “Acanthamoeba” were also used, to search for any cellular homolog (which were not found). Multiple sequence alignments were performed by Clustal Omega [27] at the EMBL-EBI web server [28], as described before [10].

## 7 Discussion, Summary and Outlook

The central conclusion in this short review is that the cyclophilin (CYN) clades of the two genera in the *Mimivirales* order of giant viruses, namely *Klosneuvirus* and *Mimivirus*, possess distinctive differences. Whereas the Klosneuviral CYNs resemble the classical CYN family, the mimiviral CYNs are unique in multiple ways, largely in line with the first mimiviral CYN characterized earlier [23]. The unique sequence features that distinguish the mimiviral CYNs from canonical CYNs are the lack of PPIase residues and activity, and major sequence insertions in the central catalytic region. Of note, the relationship between PPIase activity and chaperone function of CYN has been debated, since PPIase-defective CYNs may exhibit wild type levels of protein folding activity [9]. For example, recombinant human CYNA, in which the catalytically important residues R55, F60, Q63, and H126 (Fig. 3) were changed to Ala, were still able to suppress the aggregation of arginine kinase, a classic CYNA folding substrate [9]. It will be interesting to determine if the mimiviral CYNs also possess chaperone function in the absence of PPIase activity. Overall, the biological relevance and evolutionary origin of the mimiviral CYNs currently remain a mystery.

Outside of their catalytic cores, the larger members of the cellular CYN family often contain diverse domains of protein-protein interaction, which include, but are not limited to: WD40, TPR, RRM and Zn-finger [2, 29]. Beyond the catalytic central region, the klosCYNs have C-terminal extensions, while the mimiCYNs are extended towards the N-terminus. Neither group, however, contains any recognizable accessory or interaction domain. The peptide insertions, due to their location in the flexible, looped-out regions, may also play a major role in interaction with other proteins. It is to be noted that mimiCYN is neither the first nor the only example of peptide insertions in CYN, and that the loop areas appear to be hotspots for such insertions. For example, when compared to human CYNA, a large number of cellular cyclophilins are found to contain 7–8 amino acid long insertions in the same loop [30] that harbors an insert in essentially the same position as insert #1 of mimiCYN (Fig. 3). Such CYNs include orthologs from diverse organisms [30], ranging from (organism name followed by GenBank number) a freshwater cyanobacterium (*Synechocystis sp.* P73789.1), unicellular protists (*C. elegans* P52011.1*, T. cruzi* O76990*, B. malayi* Q27450.1*, Onchocerca volvulus* AAD09564.1), and plants (*A. thaliana* P34790.1*, Zea mays* P21569.1), to large mammals (*Bos taurus* P26882.6). Of these, the *T. cruzi* CYN also contains another insertion where mimiCYN insert #2 is located.

In view of the many novel features of the mimiCYN family, as discussed above, I embarked on an intense search for homology with the hope of gaining an insight into their origin and/or function. However, use of whole or overlapping parts of the mimiCYNs as query sequences and various BLAST programs and parameters failed to retrieve any significant homolog from all non-mimivirus sequence databases. Pathogens and parasites often function as gene transmission vectors through genetic recombination, and this has also been implicated for giant viruses [19, 20]. Specifically, the amoeba serves as the host for many mimiviruses, both in nature and in the laboratory, and therefore, efforts were made to determine if the mimiCYN genes may have been derived from various lineages of amoeba, such as *Acanthamoeba*, *Amoeba*, *Dictyostelium*, and *Entamoeba*. Again, no obvious host CYN homolog resembling the unique features of mimiCYN could be found; in other words, the amoeba cyclophilins were found to possess the canonical features. One search returned the GenBank entry WP_082173706.1, described as a hypothetical, partial protein from the bacterium *Microvirga massiliensis* (the corresponding whole genome shotgun sequence is NZ_CAHM01000427.1). I ignored this entry because it was 100% identical to the Powai lake megavirus CYN (ANB50819.1), which may have resulted from contamination during the sequencing of the *Microvirga* genome [31] or a mix-up during annotation. Finally, the nucleotide sequences flanking the CYN gene on either side in the mimiviral genomes were used to search for similar sequences in the host genomes mentioned above, since such repeats could promote homologous recombination, which in mimiviruses, may be catalyzed by site-specific homing endonucleases; however, no significant sequence repeat was found. On a similar note, several mimiviruses encode amino acyl tRNA synthetases, but phylogenetic studies did not find any close homologs in the host amoeba [18]. Overall, the roots of mimiCYNs and their inserts, therefore, remain a mystery, probably owing their origin to multiple sources [19, 20]. Nonetheless, the high similarity of the mimiCYN sequences among themselves may indicate lateral CYN gene transfer from one mimivirus to another, accompanied by selection for minor changes to suit the needs of the individual viruses.

## Conflict of Interest

None.

## Funding

No external funds were used for these studies. The author's personal fund was used to pay for publication costs and open access fee.

## Acknowledgements

None.
